# Connections among Land Use, Water Quality, Biodiversity of Aquatic Invertebrates, and Fish Behavior in Amazon Rivers

**DOI:** 10.3390/toxics10040182

**Published:** 2022-04-07

**Authors:** Rodrigo Silva de Sousa, Gilmar Clemente Silva, Thiago Bazzan, Fernando de la Torre, Caroline Nebo, Diógenes Henrique Siqueira-Silva, Sheila Cardoso-Silva, Marcelo Luiz Martins Pompêo, Teresa Cristina Brazil de Paiva, Flávio Teixeira da Silva, Daniel Clemente Vieira Rêgo da Silva

**Affiliations:** 1 Institute of Xingu Studies, Federal University of Southern and Southeastern Pará, São Félix do Xingu 68380-000, Pará, Brazil; rodrigo.silva@unifesspa.edu.br; 2 Postgraduate Program in Environmental Technology, Fluminense Federal University, Volta Redonda 27255-125, Rio de Janeiro, Brazil; gilmarcs@id.uff.br; 3 Earth Observation and Geoinformatics Division, National Institute for Space Research, São José dos Campos 12227-900, São Paulo, Brazil; thiago.bazzan@inpe.br; 4 Institute of Ecology and Sustainable Development, National University of Lujan, Buenos Aires 6700, Argentina; flatorre_ar@yahoo.com; 5 Institute of Studies of the Humid Tropics, Federal University of Southern and Southeastern Pará, Xinguara 68555-016, Pará, Brazil; carolnebo@unifesspa.edu.br; 6 Institute of Health and Biological Studies, Federal University of Southern and Southeastern Pará, Marabá 68507-590, Pará, Brazil; diogenessilva@unifesspa.edu.br; 7 Institute of Oceanography, University of São Paulo, São Paulo 05508-120, São Paulo, Brazil; she.cardosos@gmail.com; 8 Department of Ecology, University of São Paulo, São Paulo 05508-090, São Paulo, Brazil; mpompeo@ib.usp.br; 9 Department of Biotechnology, Engineering School of Lorena, University of São Paulo, Lorena 12602-810, São Paulo, Brazil; teresapaiva@usp.br (T.C.B.d.P.); flaviots@usp.br (F.T.d.S.); 10 Institute of Exact Sciences, Federal University of Southern and Southeastern Pará, Marabá 68507-590, Pará, Brazil

**Keywords:** aquatic biodiversity, fish habitats, habitat fragmentation, non-forced exposure, water pollution

## Abstract

Rivers in the Amazon have among the greatest biodiversity in the world. The Xingu River, one of the tributaries of the Amazon River, has a length of 1640 km, draining 510,000 km^2^ in one of the most protected regions on the planet. The Middle Xingu region in Brazil has been highly impacted by mining and livestock farming, leading to habitat fragmentation due to altered water quality. Therefore, comparing two rivers (the preserved Xingu River and the impacted Fresco River) and their confluence, the aims of the present study were to (1) assess the land uses in the hydrographic basin; (2) determine the water quality by measurements of turbidity, total solids, and metals (Cd, Cu, Fe, Mn, Pb, Zn, and Hg); (3) compare the zooplankton biodiversity; and (4) to evaluate the avoidance behavior of fish (*Astyanax bimaculatus*) when exposed to waters from the Xingu and Fresco Rivers. Zooplankton were grouped and counted down to the family level. For the analysis of fish avoidance, a multi-compartment system was used. The forest class predominated at the study locations, accounting for 57.6%, 60.8%, and 63.9% of the total area at P1XR, P2FR, and P3XFR, respectively, although since 1985, at the same points, the forest had been reduced by 31.3%, 25.7%, and 27.9%. The Xingu River presented almost 300% more invertebrate families than the Fresco River, and the fish population preferred its waters (>50%). The inputs from the Fresco River impacted the water quality of the Xingu River, leading to reductions in local invertebrate biodiversity and potential habitats for fish in a typical case of habitat fragmentation due to anthropic factors.

## 1. Introduction

The Xingu River is one of the largest tributaries of the Amazon River, originating in Mato Grosso state and crossing the state of Pará. It has a length of 1640 km, draining 510,000 km^2^ in one of the most protected regions on the planet, composed of a mosaic of preserved areas. There are approximately 20 indigenous lands and conservation units, forming a corridor of 280,000 km^2^ [1,2]. Knowledge about the biodiversity of the Xingu River is still limited, although studies have highlighted its great potential for the region [1]. According to Jézéquel et al. [3], it is estimated that approximately 821 species of fish inhabit the basin, 73 of them being endemic. However, this diversity is exposed to the constant impacts of different land uses in the region [4].

One of the main problems in the basin, including a southeast tributary of the Xingu River (Fresco River), is the extraction of wood, with implementation of livestock, agriculture, and mining activities. Changes in riparian forest can modify the quality and quantity of litter produced [5]. This material is an important energy source for heterotrophic organisms [6], and changes in the input of organic matter can influence the assemblages of aquatic organisms, modifying the distribution and abundance of different taxa [7]. According to Ilha et al. [8], deforestation along the Xingu River’s streams significantly changed their natural and functional diversity. Furthermore, riparian vegetation has a major influence on fluvial geomorphology by affecting flow resistance, soil mechanical strength in ravines, sediment storage, bed stability, and channel morphology [9], thus preventing silting and the entry of particulate matter into the water. Between 2000 and 2010, the Xingu hydrographic basin experienced major changes in land cover. Approximately 12% of the hydrographic basin, 19,000 km^2^, was deforested up to the year 2000 [10], and it is considered one of the most important agricultural regions in Brazil, accounting for 13% of national production of soybean, exported to Asia and Europe [11]. Furthermore, after the removal of vegetation, other activities are carried out at these sites, such as mining, which disturbs the soil and facilitates its entry into the water column, and conversion to pastures, which hinders the regeneration of vegetation, in addition to making the soil more compacted and infertile [12,13]. When soil margins become exposed, the direct impact of rainwater causes the detachment of particles, known as the splash effect. Surface runoff, due to its abrasive forces, removes soil particles and transports them into water bodies, changing the quality of the medium, reducing water transparency, interfering with photosynthetic processes, and unbalancing the food chain [13]. The reduced visibility affects the foraging of various fish, with the populations of visually oriented organisms decreasing in rivers impacted by anthropogenic sediment [14,15], culminating in the loss of fish diversity [16].

The fish species *Astyanax bimaculatus* (Linnaeus 1758) (twospot *Astyanax*), a member of the Characidae family, has a wide distribution in the Brazilian northeast region and is the main constituent of the diets of other local fish, including *Oligosarcus longirostris* and *Hoplias malabaricus* [17]. The adaptive plasticity of this species allows its successful reproduction and survival in the most varied habitats of this region of the Amazon, including lakes, reservoirs, streams, swamps, and rivers [18]. For this reason, *A. bimaculatus* has been used as a suitable model for toxicology [19,20], as well as in environmental impact studies and environmental safety [21,22]. In order to assess water quality, standardized ecotoxicological tests are employed, in which organisms from various taxa can be used as bioindicators. Ecotoxicology is concerned with detecting adverse effects at an ecosystem level and protecting individual species [23]. The responses measured in these tests are based on survival, growth, development, and reproduction, with the tests being standardized to improve reliability, reproducibility, and comparability across tests and substances [24]. However, the concentrations used are often not environmentally relevant, exceeding those found in natural environments, and organisms are exposed in a confined manner during the assays. These tests assume that organisms in natural ecosystems are forced to remain exposed to contaminants, without the possibility of escape [25]. A new and more realistic approach has been used in recent years, where bioindicator organisms are exposed in a multi-compartment system [26,27,28,29,30], enabling exposure to different contamination scenarios, with the objective of identifying areas that are more attractive or repellent. Such experiments are a suitable way to understand the environmental risks caused by the repellent characteristics of contaminants [31].

In order to investigate the quality of the waters of the Xingu River and its Fresco River tributary (both belong to the same sub-basin) and the effect of their mixing, the aims of the present study were as follows: (1) to assess the land uses in the hydrographic basin; (2) to investigate the water quality using measurements of turbidity, total solids, and metals (Cd, Cu, Fe, Mn, Pb, Zn, and Hg); (3) to compare the zooplankton biodiversity; and (4) to evaluate the avoidance behavior of fish (*A. bimaculatus*). Contaminated rivers can exert a strong influence on the rivers into which they flow, which is an important factor in determining the pattern of habitat selection by organisms after mixing of the waters. Therefore, it was hypothesized that the Fresco River, due to the impacts of mining on its waters, could affect the patterns of invertebrate biodiversity and fish habitat selection after its confluence with the Xingu River.

## 2. Materials and Methods

### 2.1. Study Area

**Figure 1:**
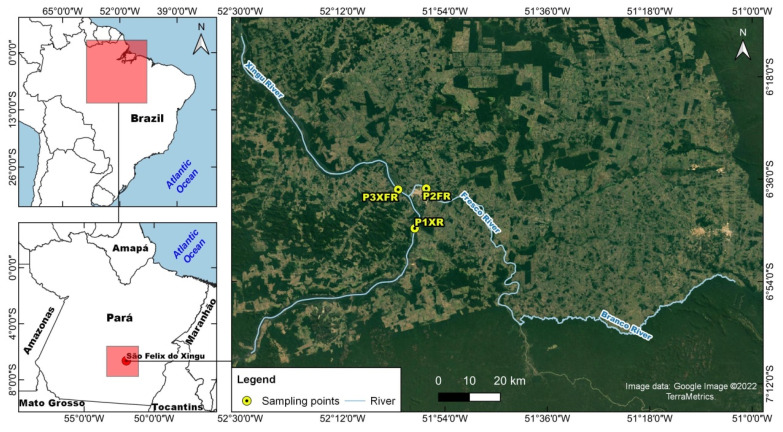
Study area and locations of the three sites for collection of water and aquatic invertebrates. Three rivers are shown: the Xingu River and the Fresco River, in the municipality of São Félix do Xingu, Pará state, and the Branco River, in the municipality of Ourilândia do Norte, Pará state, Brazil. Source image data: Google Image ©2022 TerraMetrics.

The study area corresponded to the sections of the Xingu and Fresco Rivers in the state of Pará, Brazil, within the coordinates 6°10′12.0″ S and 7°07′48.0″ S latitude and 51°39′00.0″ W and 52°31′12.0″ W longitude (Figure 1). Water samples and invertebrate animals were collected close to the city of São Félix do Xingu (SFX), in July 2019, in the dry season, at three different sites: P1XR, upstream on the Xingu River (before the confluence with the Fresco River), at 6°44′39.33″ S/51°59′19.58″ W; P2FR, upstream on the Fresco River (before the confluence with the Xingu River), at 6°37′38.39″ S/51°57′17.67″ W; and P3XFR, after the confluence of the two rivers, at 6°37′50.18″ S/52°2′14.64″ W. The areas were selected based on the potential land use of each basin. At each sampling point, surface water samples (5 L) were obtained (in triplicate) from the first centimeters of the water column, using plastic bottles.

### 2.2. Hydrography, Land Use, and Land Cover

The mapping of land cover and use was performed using the MapBiomas Collection 6 plug-in for the QGIS geographic information system (GIS) software package. MapBiomas maps are produced from pixel-by-pixel classification of Landsat satellite images, with spatial resolution of 30 m. Collection 6 of the MapBiomas Project (http://mapbiomas.org) (accessed on 31 January 2022), released in August 2021, covers the period from 1985 to 2020 [32]. In QGIS, the vectorization of the banks of the Xingu and Fresco Rivers was carried out for the water class of the MapBiomas map in each study section. From the vectorization of the edges, a distance map (buffer) of 120 m was calculated for both edges to quantify the classes of land cover and use [33]. Land use and vegetation cover on the banks of the Xingu and Fresco Rivers were represented by four classes: forest, non-forest natural formations, agriculture and livestock, and non-vegetated areas.

### 2.3. Water Quality

For all the following analyses, only P1XR and P2FR were studied. The analyses of seven metals (Cd, Cu, Fe, Mn, Pb, Zn, and Hg) were performed based on the APHA method [34] by inductively coupled plasma optical emission spectrometry (ICP-OES), using an ICPE-9000 multitype instrument (Shimadzu, Kyoto, Japan) operated with plasma gas flow rate of 10 L∙min^−1^, RF power of 1200 W, and nebulizer gas flow rate of 0.7 L∙min^−1^. Before analysis, the samples were filtered to remove solids.

The gravimetric method was used for the analysis of total solids. Aliquots of approximately 200 mL of the samples were added to metal flasks and placed in an oven at 105 °C to 180 °C. The following calculation was used to obtain the data: (P2 − P1)/V × 1000, where P1 is the weight of the oven-dried beaker without the sample, P2 is the weight of the oven-dried beaker with the sample, and V is the volume of the sample used for evaporation [35]. For the analysis of turbidity, a bench turbidimeter was used (Model DLT-WV, Del Lab).

### 2.4. Zooplankton Biodiversity Analysis

Samples from P1XR, P2FR, and P3XFR were used for the zooplankton biodiversity analysis. Nets with diameter of 30 cm and mesh with 200 μm holes were used to collect zooplankton from the surface water [36]. The samples were preserved in 4% formaldehyde solution, with fixing using 5 G·L^−1^ sodium bicarbonate [37]. The organisms were identified with specific keys [38], up to the classification of families (morphotypes). The total number of families represented the diversity of the organisms, while the abundance considered the number of individuals per family.

### 2.5. Experimental Methods for the Avoidance Tests

#### 2.5.1. Test Organisms

Juveniles of the native fish species *A. bimaculatus* (2–3 months of age, approximately 1.5 cm long) were acquired from a local fish farm in the municipality of São Félix do Xingu. In the laboratory, the fish were kept in well water, with continuous aeration, average temperature of 25 ± 3 °C, and feeding twice daily (with Tetramin commercial diet: 46% crude protein and 11% crude fat) until apparent satiety. These conditions were maintained during two weeks, for acclimation before the experiments, following the OECD guidelines [39].

#### 2.5.2. Avoidance Tests

First, a control test (quadruplicates) was carried out with filtered well water in order to demonstrate that the environment and the avoidance system did not influence the distribution of fish, and then, water samples from P1XR, P2FR, and P3XFR were used for the avoidance tests (quadruplicates) (at the lab scale). Both tests were performed in a multi-compartment system constructed from borosilicate glass [26]. The system had 6 chambers, where chambers C1 and C2 contained water from P1XR, chambers C3 and C4 contained water from P3XFR (downstream of the confluence of the rivers), and chambers C5 and C6 contained water from P2FR, consequently forming a gradient from the most preserved river (Xingu) to the most impacted river (Fresco). Since the confluence sample (P3XFR) contained water from both rivers, it was considered an intermediate environment. The tests were started by placing 3 *A. bimaculatus* fish in each chamber, totaling 18 animals in the system. Measurements of conductivity and pH were made at the beginning (0 h) and end (3 h) of the experiment. The tests were performed in quadruplicate, in the dark, at a temperature of 25 ± 3 °C.

### 2.6. Statistical Analysis

One-way ANOVA (*p* < 0.05), followed by the Tukey test, was employed to analyze the random distribution of fish in the control (number of individuals among each chamber), and the distribution of fish in the avoidance tests (expressed as the distribution of organisms (number of individuals), among each treatment with river water: Average of C1–C2 (P1XR), C3–C4 (P3XFR), and C5–C6 (P2FR)). Principal component analysis (PCA) was applied to identify the most important variables in the study area and the relationships among all the variables.

## 3. Results

### 3.1. Hydrography, Land Use, and Land Cover

**Figure 2:**
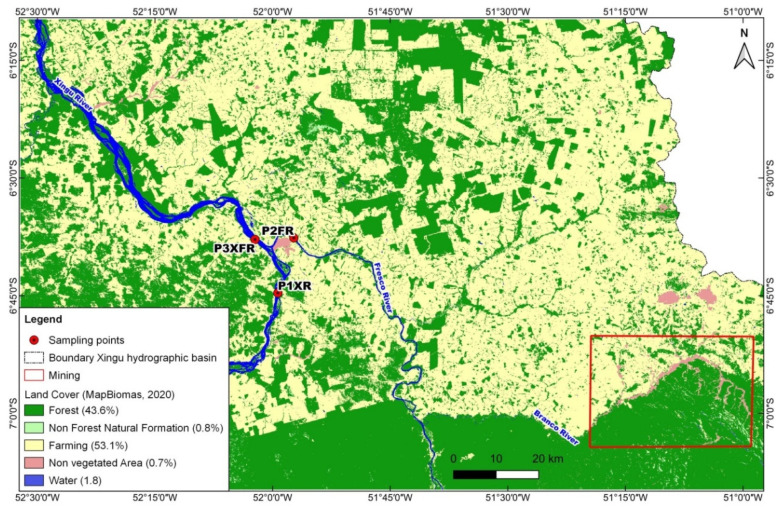
Map showing the spatial distribution of land use classes and vegetation cover in the study area in the municipality of São Félix do Xingu, Pará state, Brazil. The red rectangle in the lower right-hand corner indicates an extensive mining area near the Branco River, a tributary of the Fresco River.

In the hydrographical assessments, each section analyzed had a total length of 89 km. In the study region, the agricultural class predominated along the margins, occupying 53.15% of the total area. The forest class occupied 43.61% of the total area, the non-forest natural formations class occupied 0.81%, and the non-vegetated area class occupied 0.68%. Figure 2 shows the percentages for the land use and vegetation cover classes in the study area. Since 1985, vegetation in this area had been reduced by 50.69% (Table S1–S4, Supplementary Materials).

The forest class predominated at the study locations, accounting for 57.6%, 60.8%, and 63.9% of the total area at P1XR, P2FR, and P3XFR, respectively. Agriculture and livestock (farming) accounted for 37.7%, 33.9%, and 33.3% of the total area at P1XR, P2FR, and P3XFR, respectively. Since 1985, the forest has been reduced by 31.3%, 25.7%, and 27.9% at P1XR, P2FR, and P3XFR, respectively (Table 1).

### 3.2. Water Quality

Only the metals Mn and Zn were detected in the water samples (*n* = 3), with average values of 0.67 ± 1.15 µg·L^−1^ and 15.33 ± 5.51 µg·L^−1^, respectively, at P1XR, and 4.0 ± 1.00 µg·L^−1^ and 31.67 ± 30.24 µg·L^−1^, respectively, at P2FR (Table S5, Supplementary Material).

The total solids concentrations (*n* = 3) were 25.25 ± 0.17 mg·L^−1^ and 147.90 ± 13.28 mg·L^−1^ at P1XR and P2FR, respectively (Table S6, Supplementary Material). The turbidity values (*n* = 3) were 3.35 ± 0.17 UT at P1XR and 116.44 ± 1.84 UT at P2FR (Table S7, Supplementary Material).

**Figure 3:**
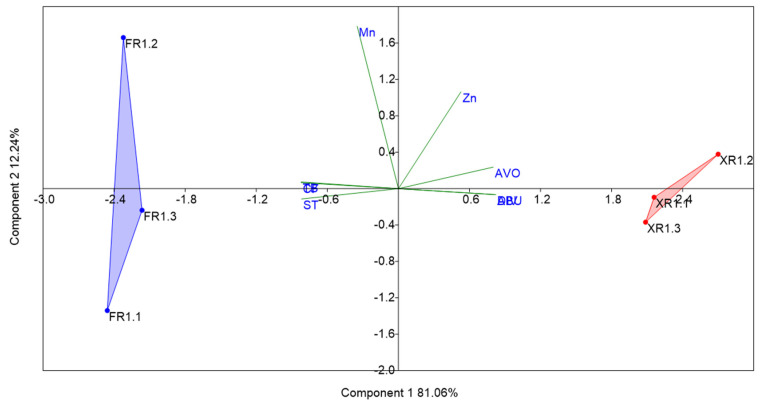
PCA components plot (based on the correlation matrix) for the waters of the Xingu River (XR) and the Fresco River (FR), considering the following variables: Mn, Zn, avoidance (AVO), total solids (TS), turbidity (TB), area with forest (FOR), aquatic invertebrate abundance (ABU), and aquatic invertebrate diversity (DIV).

The PCA results (Figure 3) showed that the two locations were distinct from each other. Based on the correlation matrix, axis 1 explained 81.06% of the total variance. The eigenvalues revealed strong relationships among the avoidance (AVO), abundance (ABU), and diversity parameters (DIV) (Tables S8 and S9, Supplementary Material). This interaction is strongly related to P1XR, which is considered a more preserved river. The total solids (TS) and Turbidity (TB) parameters had a strong relationship with P2FR, being inversely proportional to the other parameters described. Since it was not possible to analyze the water quality at the confluence of the rivers (P3XFR), information for this section was not included in the PCA.

### 3.3. Zooplankton Biodiversity Analysis

At P1XR, counting revealed 48 different organisms (families/genera), with an abundance of 1361 individuals. At P2FR, there were 15 different organisms, with an abundance of 262 individuals, while at P3XFR, there were 21 different organisms, with an abundance of 330 individuals (Table S10, Supplementary Materials).

### 3.4. Avoidance Tests

**Figure 4:**
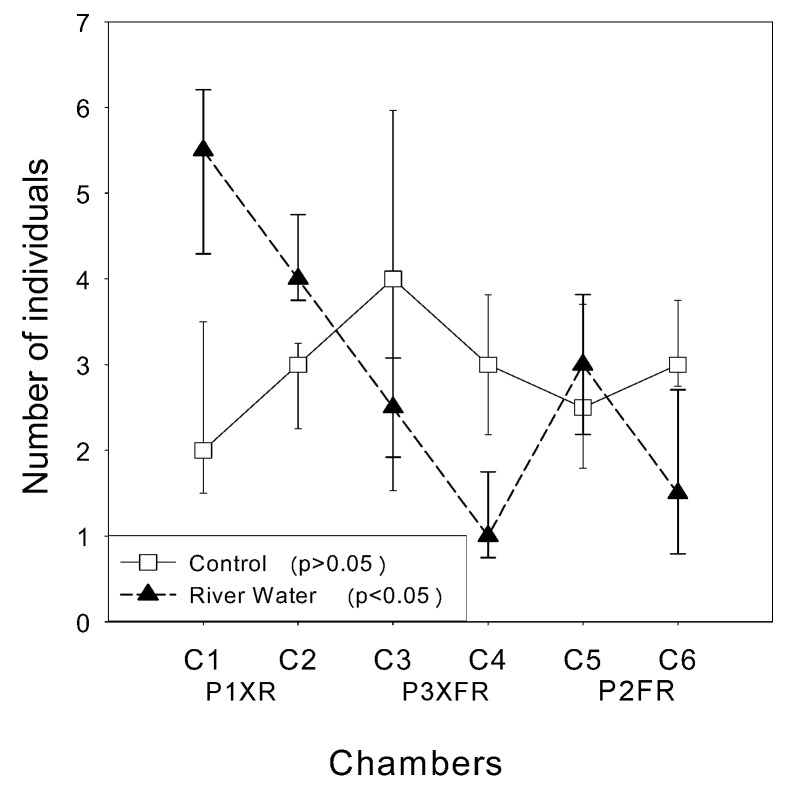
Distributions of *A. bimaculatus* (twospot *Astyanax*) fish in the control assay (using well water) and in the assay with exposure to river water (C1 and C2 = P1XR; C3 and C4 = P3XFR; C5 and C6 = P2FR).

In the control test, the data indicated a random distribution (Figure 4; Tables S11 and S12, Supplementary Materials), with no differences in the distribution of fish among compartments (*p* > 0.05).

In the treatments with river water, there was a pattern of the organisms avoiding the P2FR and P3XFR waters (Figure 4 and Table 2; Table S13–S15, Supplementary Materials), with over 50% (number of individuals converted to percentage) of the fish being found in the P1XR water.

## 4. Discussion

Conserving natural vegetation cover is of critical importance for maintaining the ecological integrity and hydrological characteristics of large river basins. Recent estimates indicate that more than 700,000 km^2^ of the Brazilian Amazon have already been deforested. From 1985 to 2015, 60,900 km^2^ of forest were converted to pasture, corresponding to 29% of the total area of the basin [40,41], with the major activities in the region being livestock farming and mining. In the São Félix do Xingu region, the data showed a reduction in forest, which occupied around 56.39% of the area analyzed (including riparian forest). Considering that in 1985, the forest occupied 94.3% of the area, 50.69% of the forest has so far been removed, which is highly concerning when compared to the 20% deforestation rate for the Amazon as a whole [42,43]. The influence of these changes in the basin on water quality extends beyond the region of the main channel of the river and its banks. Tributaries exert enormous pressure on central water bodies such as the Xingu River. This river receives the adversely impacted waters of the Fresco River, which in turn receives the waters of the Branco River, in a region where there is great pressure from mining. In this case, it was observed that despite having vegetation cover similar to that of the Xingu River, the quality of its waters did not reflect the degree of preservation of its banks.

The water quality analysis detected the two metals Mn and Zn in both the Xingu River and the Fresco River. The levels of these elements were 5.97 and 2.06 times higher, respectively, at P2FR, compared to P1XR. The application of PCA showed that P1XR had stronger relationships with invertebrate abundance and diversity, as well as avoidance (fish attraction). On the other hand, there were inversely proportional relationships with turbidity and total solids. These last parameters showed a strong relationship with P2FR. Horb et al. [44] reported Mn and Zn concentrations of 17 and 19 µg·L^−1^, respectively, in the Madeira River and its tributaries. The Madeira River is a white-water river (muddier water) with a high load of suspended sediments [45], analogous to the P2FR water in the present case. The presence of these metals was related to their natural occurrence in Amazon soils and their entry into water bodies by means of surface runoff. The concentrations depend on the source of the rivers and the geological formations related to the channel through which the river passes [46]. At P2FR, the water included inputs from the Branco River, affected by intense mining activity in the region (Figure 2, indicated by the red rectangle). The elimination of riparian vegetation and disturbance of the soil leads to greater entry of chemical elements into the water body [47], which could explain the difference in the concentrations of the metals between P1XR and P2FR.

The total solids (TS) and turbidity results also showed a considerable difference between the two water bodies. At P2FR, TS was 5.85 times higher than at P1XR. Rakotondrabe et al. [48] showed that mining activity in Bétaré-Oya (East Cameroon) led to high levels of TS in the river waters, with an average of 283.58 mg·L^−1^. This same pattern appeared to occur at P2FR. Khan et al. [49] showed the impact of some tributaries in India, which could reduce the quality of the water bodies into which they flowed.

Cajado et al. [50] reported low turbidity values of around 1.25 ± 0.05 UT for the waters of the Tapajós River, a clear water river, similar to the river at P1XR. The turbidity at P2FR was 34.75 times higher than at P1XR. It is known that the reduction in light penetration in turbid waters leads to lower phytoplankton biomass, which influences the secondary productivity of the water body [51].

There are several factors necessary for the maintenance of biodiversity. Preserved rivers are expected to present a diversity of niches and abundant resources, maintaining a fluvial continuum and tending to be more balanced and complex, with potential for intra- and interspecific relationships [6]. Furthermore, benthic invertebrates, fishes, zooplankton, and periphyton all respond to the temporal variability of the river system, which depends on the regional hydrological cycle [52,53]. The zooplankton community is only one component in an extensive system that involves all the benthic, fish, periphyton, and phytoplankton communities. Significant differences occur among aquatic communities found at distinct stages of the hydrological regime [54]. According to the results obtained for the diversity (morphotypes) of aquatic invertebrates, the water at P1XR had 3.2 and 2.28 times higher invertebrate diversity, compared to P2FR and P3XFR, respectively. The invertebrate abundance at P1XR was more than double the combined abundance for P2FR and P3XFR. In this case, the Xingu River, at least in the area before the confluence, had the necessary environmental conditions to maintain the quality standard of its waters, leading to greater biodiversity of aquatic invertebrates, compared to its tributary, the Fresco River.

In more impacted rivers, the diversity of organisms can be drastically reduced, leading to ecosystem imbalance, as observed for P2FR. After the confluence of the two rivers, at P3XFR, there was a pattern, at least locally, of decreasing abundance and diversity of organisms. In this case, there was evident fragmentation of habitat caused by the entry of impacted water into a river that was expected to be more conserved. The impacts of mining were reported by Ribeiro et al. [4], who described the Fresco River, in the same area as P2FR, as having yellowish waters, large amounts of suspended organic matter, and foamy material on the surface. The study also found high concentrations of Ni and Cr in the river sediment. This decrease in water quality could be one of the factors responsible for the reduction in invertebrate biodiversity in this water body.

The quality of water in a water body directly affects biodiversity and habitat selection by the organisms present. In impacted regions, metals accumulated in sediments pass through the trophic chain to fish. This exposure typically leads to ecotoxicological adverse effects [55]. In given regions of a water body, the process of habitat fragmentation can occur due to several environmental factors, including anthropogenic pollution that introduces barriers to the movement of organisms, reducing or preventing the presence of certain species [56]. Impacts on fauna can be severe, especially when they occur at distances beyond those to which organisms are able to disperse [57].

The results obtained in the avoidance tests showed the preference of the fish for the water from P1XR (*p* < 0.05). In the experimental design employed, 51.85% of the population of *A. bimaculatus* remained in the Xingu River water, while only 20.37% remained in the water resulting from the mixing of the two rivers (P3XFR). Extrapolating to the real environment, there would be 2.5 times more fish in the Xingu River upstream of the confluence, compared to downstream of it, reflecting a clear impact on the water quality of the main water body. Hence, the confluence of the rivers could be responsible for habitat fragmentation downstream in the Xingu River, consequently reducing the potential for the dispersion of the *A. bimaculatus* population downstream. Chemical barriers can act as a potential element in habitat fragmentation, contributing to the restriction of the movement of fish in water bodies, as reported by Araújo et al. [58]. Another study showed that poor water quality acted as a barrier to upstream migration of *Alosa fallax fallax* in the River Scheldt, West Europe [59]. Finally, it was observed that the Fresco River exerted great negative pressure on the Xingu River, due to the low quality of its waters. The loss of organisms, due to avoidance, can have important ecological implications for the structure and functioning of ecosystems. Avoidance, according to Araujo et al. [31], can create imbalances in communities, affecting interactions among organisms and even biogeochemical cycles (such as energy flow and nutrient cycles).

## 5. Conclusions

There was a clear water quality difference between the Xingu and Fresco Rivers. This was due to the better quality of the water of the Xingu River, where more aquatic invertebrates were found and for which the avoidance tests showed greater preference of *A. bimaculatus*, in terms of habitat selection. In the confluence area, the water of the Fresco River affected the water quality of the Xingu River, reducing the potential for fish dispersion, as well as invertebrate biodiversity, downstream in the Xingu River. This constituted a typical case of habitat fragmentation caused by anthropic factors, more specifically mining.

## Figures and Tables

**Table 1:** Spatial distribution of land use classes and vegetation cover (from 1985 to 2020) in the study area (in%) in the municipality of São Félix do Xingu, Pará state, Brazil, from the P1XR, P2FR, and P3XFR locations.

Class	P1XR 1985	P1XR 2020	P2FR 1985	P2FR 2020	P3XFR 1985	P3XFR 2020
Forest	88.9	57.6	86.5	60.8	91.8	63.9
Non-forest natural formation	5.0	4.2	4.0	3.6	2.9	2.6
Farming	5.6	37.7	8.2	33.9	5.1	33.3
Non-vegetated Area	0.5	0.6	1.4	1.7	0.1	0.1
Total	100	100	100	100	100	100

**Table 2:** Percentage distributions of *A. bimaculatus* (twospot *Astyanax*) according to chamber and treatment, using the river water samples from the P1XR, P2FR, and P3XFR locations.

Chambers	Chamber (%)	Treatment (%)
C1 (P1XR)	27.78	51.85
C2 (P1XR)	24.07	
C3 (P3XFR)	12.96	20.37
C4 (P3XFR)	7.40	
C5 (P2FR)	16.66	27.77
C6 (P2FR)	11.11	

## Data Availability

Data are available as Supplementary Material.

## Supplementary Materials

The following are available online at https://www.mdpi.com/article/10.3390/toxics10040182/s1: Table S1: Comparison between 1985 and 2020 of land use classes and vegetation cover in the study area, in the municipality of São Félix do Xingu, Pará state, Brazil, Table S2: Comparison between 1985 and 2020 of land use classes and vegetation cover at the P1XR site, in the municipality of São Félix do Xingu, Pará state, Brazil, Table S3: Comparison between 1985 and 2020 of land use classes and vegetation cover at the P2FR site, in the municipality of São Félix do Xingu, Pará state, Brazil, Table S4: Comparison between 1985 and 2020 of land use classes and vegetation cover at the P3XFR site, in the municipality of São Félix do Xingu, Pará state, Brazil, Table S5: Concentrations of Mn and Zn (µg·L^−1^) in water samples from P1XR and P2FR, Table S6: Concentrations of total solids (mg·L^−1^) in water samples from P1XR and P2FR, Table S7: Turbidity (UT) of water samples from P1XR and P2FR, Table S8: Principal component analysis (based on the correlation matrix), Table S9: Principal component analysis loadings, considering the following variables: Mn, Zn, avoidance (AVO), total solids (TS), turbidity (TB), area with forest (FOR), aquatic invertebrate abundance (ABU), and aquatic invertebrate diversity (DIV), Table S10: Numbers of zooplankton collected, according to family (morphotypes), showing the abundance and diversity of the organisms, Table S11: Distribution of *A. bimaculatus* in the control test with well water, Table S12: ANOVA applied to the distribution of the organisms in the control test, Table S13: Distribution of *A. bimaculatus* in the test with water from P1XR, P3XFR, and P2FR, Table S14: One-way ANOVA applied to the distribution of the organisms in the test with water from P1XR, P3XFR, and P2FR, Table S15: Tukey’s test pairwise comparisons (*p* < 0.05) for the distribution of the organisms in the test with water from P1XR (C1 and C2), P3XFR (C3 and C4), and P2FR (C5 and C6) in the avoidance system.
