# Supplementary material for: Connections among Land Use, Water Quality, Biodiversity of Aquatic Invertebrates, and Fish Behavior in Amazon Rivers
Source: Toxics. 2022 Apr 7;10(4):182. doi: 10.3390/toxics10040182 (PMC9031983; doi:10.3390/toxics10040182)
Supplement: Supplementary file 1 [file toxics-10-00182-s001.zip › toxics-1666330-supplementary.pdf]

toxics MDPI

Supplementary Materials: Connections among land use, water quality, biodiversity of aquatic invertebrates, and fish behavior in Amazon rivers

Rodrigo Silva de Souza, Gilmar Clemente Silva, Thiago Bazzan, Fernando de la Torre, Caroline Nebo, Diógenes Henrique Siqueira-Silva, Sheila Cardoso-Silva, Marcelo Luiz Martins Pompêo, Teresa Cristina Brazil de Paiva, Flávio Teixeira da Silva and Daniel Clemente Vieira Rêgo da Silva

Table S1. Comparison between 1985 and 2020 of land use classes and vegetation cover in the study area, in the municipality of São Félix do Xingu, Pará state, Brazil.

STUDY AREA				
**Class**	**1985 (km²)**	**1985 (%)**	**2020 (km²)**	**2020 (%)**
Forest	16,997.3	94.3	7860.7	43.61
Non-forest natural formation	183.5	1.02	145.3	0.81
Farming	521.1	2.89	9578.7	53.15
Non-vegetated area	7.9	0.04	122.6	0.68
Water	314.4	1.74	316.3	1.75
Total	18,024.2	100	18,023.6	100.0

Table S2. Comparison between 1985 and 2020 of land use classes and vegetation cover at the P1XR site, in the municipality of São Félix do Xingu, Pará state, Brazil.

Class	P1XR 1985 (km²)	P1XR 1985 (%)	P1XR 2020 (km²)	P1XR 2020 (%)
Forest	24.7	88.9	16.3	57.6
Non-forest natural formation	1.4	5.0	1.2	4.2
Farming	1.6	5.6	10.7	37.7
Non-vegetated area	0.1	0.5	0.2	0.6
Water				
Total	27.8	100	28.3	100

Table S3. Comparison between 1985 and 2020 of land use classes and vegetation cover at the P2FR site, in the municipality of São Félix do Xingu, Pará state, Brazil.

Class	P2FR 1985 (km²)	P2FR 1985 (%)	P2FR 2020 (km²)	P2FR 2020 (%)
Forest	23.2	86.5	16.8	60.8
Non-forest natural formation	1.1	4.0	1.0	3.6
Farming	2.2	8.2	9.4	33.9
Non-vegetated area	0.4	1.4	0.5	1.7
Water				
Total	26.8	100	27.7	100

Table S4. Comparison between 1985 and 2020 of land use classes and vegetation cover at the P3XFR site, in the municipality of São Félix do Xingu, Pará state, Brazil.

Class	P3XFR 1985 (km^2)	P3XFR 1985 (%)	P3XFR 2020 (km^2)	P3XFR 2020 (%)
Forest	26.9	91.8	19.0	63.9
Non-forest natural formation	0.9	2.9	0.8	2.6
Farming	1.5	5.1	9.9	33.3
Non-vegetated area	0.0	0.1	0.0	0.1
Water				
Total	29.3	100	29.8	100

Table S5. Concentrations of Mn and Zn (μg.L^{-1}) in water samples from P1XR and P2FR.

	P1XR		P2FR	
**Replicates**	**Mn**	**Zn**	**Mn**	**Zn**
#1	0.0	21.0	5.0	9.0
#2	2.0	10.0	4.0	66.0
#3	0.0	15.0	3.0	20.0
Average	0.67	15.33	4.00	31.67
SD	1.15	5.51	1.00	30.24

*The other metals (Cd, Cu, Fe, and Pb) were not detected.

Table S6. Concentrations of total solids (mg.L^{-1}) in water samples from P1XR and P2FR.

Replicates	P1XR	P2FR
#1	25.39	163.11
#2	25.06	142.00
#3	25.30	138.60
Average	25.25	147.90
SD	0.17	13.28

Table S7. Turbidity (UT) of water samples from P1XR and P2FR.

Replicates	P1XR	P2FR
#1	3.48	117.33
#2	3.16	117.67
#3	3.4	114.33
Average	3.35	116.44
SD	0.17	1.84

Table S8. Principal component analysis (based on the correlation matrix).

PC	Eigenvalue	% variance
1	6.49	81.06
2	0.98	12.24
3	0.50	6.26

Table S9. Principal component analysis loadings, considering the following variables: Mn, Zn, avoidance (AVO), total solids (TS), turbidity (TB), area with forest (FOR), aquatic invertebrate abundance (ABU), and aquatic invertebrate diversity (DIV).

	**Axis 1**	**Axis 2**	**Axis 3**
AVO	0.38	0.11	−0.20
TS	−0.39	−0.06	0.08
TB	−0.39	0.04	0.10
Mn	0.25	0.51	0.82
Zn	−0.17	0.85	−0.48
FOR	−0.39	0.03	0.12
ABU	0.39	−0.03	−0.12
DIV	0.39	−0.03	−0.12

Table S10. Numbers of zooplankton collected, according to family (morphotypes), showing the abundance and diversity of the organisms.

	**Number of organisms**		
Family (morphotypes)	P1XR	P2FR	P3XFR
1	336	174	268
2	2	1	0
3	371	41	29
4	425	20	1
5	22	9	1
6	0	3	1
7	0	2	0
8	0	1	0
9	1	3	1
10	4	3	1
11	1	0	0
12	0	1	0
13	14	1	0
14	1	1	0
15	1	0	0
16	0	1	0
17	0	1	2
18	2	0	1
19	0	0	1
20	0	0	4
21	0	0	2
22	0	0	1
23	1	0	3
24	3	0	7
25	0	0	1
26	0	0	1
27	2	0	2
28	0	0	1
29	17	0	1
30	0	0	1
31	3	0	0

32	44	0	0
33	2	0	0
34	31	0	0
35	2	0	0
36	1	0	0
37	3	0	0
38	1	0	0
39	1	0	0
40	3	0	0
41	1	0	0
42	11	0	0
43	10	0	0
44	5	0	0
45	1	0	0
46	2	0	0
47	2	0	0
48	4	0	0
49	1	0	0
50	1	0	0
51	1	0	0
52	1	0	0
53	11	0	0
54	1	0	0
55	1	0	0
56	1	0	0
57	1	0	0
58	4	0	0
59	1	0	0
60	3	0	0
61	3	0	0
62	1	0	0
Abundance	1361	262	330
Diversity	48	15	21

Table S11. Distribution of *A. bimaculatus* in the control test with well water.

**Replicates**	**Chambers**					
	**C1**	**C2**	**C3**	**C4**	**C5**	**C6**
#1	4	3	1	3	4	3
#2	2	2	3	4	3	4
#3	2	3	6	2	2	2
#4	2	3	5	3	2	3
Mean	2	2.7	4.7	3	2.3	3.3
SD	0	0.6	1.5	1	0.6	0.6

Table S12. ANOVA applied to the distribution of the organisms in the control test.

	Sum of squares	df	Mean square	F	p (same)	F critical
Between groups	4	5	0.8	0.6	0.700596	2.772853
Within groups	24	18	1.333333			
Total	28	23				

Table S13. Distribution of *A. bimaculatus* in the test with water from P1XR, P3XFR, and P2FR.

Replicates	Chambers					
	C1	C2	C3	C4	C5	C6
	P1XR		P3XFR		P2FR	
#1	6	4	3	1	3	1
#2	6	4	3	2	2	1
#3	5	4	2	1	3	3
#4	4	5	2	1	4	2
Mean	5.0	4.3	2.3	1.3	3.0	2.0
SD	1.0	0.6	0.6	0.6	1.0	1.0

Table S14. One-way ANOVA applied to the distribution of the organisms in the test with water from P1XR, P3XFR, and P2FR.

	Sum of squares	df	Mean square	F	p (same)
Between groups:	37.75	2	18.875	21.72	0.00000771
Within groups:	18.25	21	0.869		

Table S15. Tukey's test pairwise comparisons ($p < 0.05$) for the distribution of the organisms in the test with water from P1XR (C1 and C2), P3XFR (C3 and C4), and P2FR (C5 and C6) in the avoidance system.

	diff	lwr	upr	p adj
P2FR-P1XR	−2.375	−3.549872	−1.2001279	0.0001365
P3XFR-P1XR	−2.875	−4.049872	−1.7001279	0.0000117
P3XFR- P2FR	−0.500	−1.674872	0.6748721	0.5409110
